# Report of M. Giraldes on the Employment of the Per-Chloride of Iron

**Published:** 1854-10

**Authors:** 


Report of M. Giraldes on the Employment of the Per-chloride of Iron.—M. Giraldes has published, in an interesting *Mémoire*, an account of thirty-five experiments by the injection of the perchloride of iron into the arteries of animals. These experiments were commenced in the month of April, 1853, and continued to the month of March, 1854. They prove that a few drops of the perchloride of iron, of variable density, injected into the carotid artery of a horse, coagulate the blood contained in a portion of the vessel somewhat less than an inch in length. Thus, two drops at 49° areometer of Beaumé, three drops at 30°, and six drops at 15° produce this coagulation. The changes produced upon the blood and upon the walls of the vessel by the coagulating agent vary, *cœteris paribus*, according to the density of the perchloride. Five drops at 49° mummify (*momifient*) completely the blood contained in the artery. The same quantity of the perchloride at 15° to 20° produces a sort of clot formed by blood combined with the salt of iron and by normal fibrine. But the action of the coagulating liquid is not completely exhausted upon blood; it extends to the coats of the artery. In the first case, these membranes become disorganized; they present a yellowish-brown discoloration; become thinned, horny, and in fact mortified. In the second case the membranes of the artery are modified by the action of the chemical agent, but this modification is much less intense, and does not amount to disorganization. The epithelium and the internal coat are destroyed; the middle coat having lost its contractile properties, yields to the impulse of the liquid, and dilates; its circular fibres are easily seen upon the inner surface of the vessel. This condition is accompanied by yellowish discoloration of the fibres of the middle coat, and by their adherence to the clot. It may be asserted, that the injection of the perchloride of iron into an artery is always followed by these modifications:—

1st. The formation of a clot.

2d. Modifications in the organization of the arterial tunics.

The clot offers different characters, according as produced by an injection of the perchloride at 45°, 49°, or 30°. In the first case it it compact, homogeneous, and formed in totality of altered blood; in the second, it is formed by a mixture of blood, altered by the salt of iron enveloped in a large quantity of normal fibrine. These primary clots are followed by the formation of two others, one on the side of the heart, the other towards the periphery.

The coats of the artery present very important modifications in their organization; whenever the experiment is made with perchloride at 49°, they become disorganized; they are reduced to the state of foreign bodies, and require to be cast off. But if the experiment is made with perchloride of 15° to 30°, the changes are of quite another character. The middle coat becomes hypertrophied, contracts adhesions with the clot, which it tends to encyst in the cavity of the vessel. The external coat is infiltrated by a fibrinous matter, plastic lymph, which may extend for some distance. After the first formations, there is established in the diseased structures a process of elimination and repair. The process of elimination takes place when both artery and clot are disorganized; they soften, and are cast off. These changes may extend to some distance, when the feeble connexion between the coats of the vessel and the secondary clots gives way, and fatal hæmorrhage ensues. The work of repair is established when both clot and arterial tunics are completely organized. The clot may soften without decomposing. But when the process of repair goes on favorably, it becomes encysted in the vessel, adheres intimately to the artery, and obliterates its calibre. As these phenomena proceed, both the plastic formations and the secondary clots disappear.

The injection of the perchloride of iron into an artery may, therefore, give rise to two classes of phenomena, primary and secondary.

The primary phenomena are:—

1. The formation of primary and secondary clots.
2. The infiltration of plastic lymph into the sheath of the artery and adherence of the clots.

The secondary phenomena are:—

1. The elimination of the disorganized parts.
2. Hypertrophy of the middle coat.
3. The encysting of the clots.
4. The disappearance of the secondary clots and of the plastic formations.
5. The occlusion of the artery.

The conclusions to which Dr. Giraldés arrives are:—

1. The perchloride of 49° to 45° should not be employed either in aneurisms or erectile tumors; its use may be followed by serious accidents.
2. In aneurisms and in erectile tumors, both venous and arterial, the perchloride should be either of 30° or 20° areometer of

Baumè in the proportion of 5 drops of 30°, 10 drops of 20°, for a quantity of blood equal to 3 cubic centimetres.

3. The perchloride of 45° to 46° may by used as a hæmostatic to stop deep hæmorrhages, or secondary hæmorrhages, after operations.

4. The perchloride of 25°, 20°, or 30° may be advantageously employed in hæmatic cysts, especially when they occur in the neck.

5. In certain cases, the perchloride of 30° to 49° may be employed to modify the condition of wounds in suppuration.—*Gaz. des Hôpitaux.*

